# The Influence of Severe Plastic Deformation on Microstructure and In Vitro Biocompatibility of the New Ti-Nb-Zr-Ta-Fe-O Alloy Composition

**DOI:** 10.3390/ma13214853

**Published:** 2020-10-29

**Authors:** Carmela Gurau, Gheorghe Gurau, Valentina Mitran, Alexandru Dan, Anisoara Cimpean

**Affiliations:** 1 Faculty of Engineering, “Dunărea de Jos” University of Galati, Domnească Street 47, 800008 Galati, Romania; carmela.gurau@ugal.ro (C.G.); gheorghe.gurau@ugal.ro (G.G.); 2 Department of Biochemistry and Molecular Biology, University of Bucharest, 91-95 Splaiul Independentei, 050095 Bucharest, Romania; valentinamitran@yahoo.com; 3 R&D Consultanta si Servicii, 45 Maria Ghiculeasa, 020943 Bucharest, Romania; alexandru_dan_ro@yahoo.com

**Keywords:** titanium alloy, GUM metal, microstructure, severe plastic deformation, HSHPT, bone staples

## Abstract

In this work, severe plastic deformation (SPD) of the newly designed Ti-Nb-Zr-Ta-Fe-O GUM metal was successfully conducted at room temperature using high speed high pressure torsion (HSHPT) followed by cold rolling (CR) to exploit the suitability of the processed alloy for bone staples. The Ti-31.5Nb-3.1Zr-3.1Ta-0.9Fe-0.16O GUM alloy was fabricated in a levitation melting furnace using a cold crucible and argon protective atmosphere. The as-cast specimens were subjected to SPD, specifically HSHPT, and then processed by the CR method to take the advantages of both grain refinement and larger dimensions. This approach creates the opportunity to obtain temporary orthopedic implants nanostructured by SPD. The changes induced by HSHPT technology from the coarse dendrite directly into the ultrafine grained structure were examined by optical microscopy, scanning electron microscopy and X-ray diffraction. The structural investigations showed that by increasing the deformation, a high density of grain boundaries is accumulated, leading gradually to fine grain size. In addition, the in vitro biocompatibility studies were conducted in parallel on the GUM alloy specimens in the as-cast state, and after HSHPT- and HSHPT+CR- processing. For comparative purposes, in vitro behavior of the bone-derived MC3T3-E1 cells on the commercially pure titanium has also been investigated regarding the viability and proliferation, morphology and osteogenic differentiation. The results obtained support the appropriateness of the HSHPT technology for developing compression staples able to ensure a better fixation of bone fragments.

## 1. Introduction

In spite of the extensive development and clinical use experienced by the metallic implants during the last decades, the researchers working in the field of biomaterials and biocompatibility are still seeking for the ideal biomaterial designed for a certain biomedical application [1,2,3]. Among these metals, titanium (Ti) and Ti alloys are the excellent option for orthopedic implants due to their high strength, low modulus, high corrosion resistance, light weight and excellent biocompatibility [4,5,6]. The biomedical alloy selected as an orthopedic implant is expected to not elicit cytotoxic and genotoxic effects [7], and allergic [8] and chronic inflammatory reactions in the human body [9,10]. Five categories of Ti-based alloys are described based on the microstructure obtained after processing: α alloys (hexagonally close-packed phase) and near-α alloys, duplex or two phase (α + β) alloys and near-β and β alloys (body-centered cubic phase) [6]. Near-α Ti alloys exhibit a very similar crystallographic form to alpha phase alloys at low temperature. These alloys acquire some beta phase (5–10%) by heating. Alpha-beta alloys mainly contain the α-phase and 10–30% transformed beta phase. Near beta alloys retain β-phase crystallographic form on initial cooling and secondary phases precipitate upon heating [1,2,3,4,5,6]. To note that, over the last years, there has been a growing interest in the development of the β-Ti alloys [11,12,13,14]. These alloys have been suggested to possess higher strength and lower modulus of elasticity than α and (α + β)-type alloys [15,16,17,18,19], excellent corrosion resistance [20,21,22,23,24], enhanced biocompatibility [21,25,26,27,28,29,30,31] and good formability [31,32,33].

Among these β-type Ti alloys, a new special Ti-Nb-Ta-Zr-O alloy composition (or TNTZ-O alloy) was designed and called “GUM metal” due to its remarkable set of mechanical properties like high strength, high elastic recovery, low modulus of elasticity and high plasticity at room temperature [34,35,36,37,38,39,40]. The use of GUM metal can be traced back to 2003 when Saito et al. first elaborated the Ti-23Nb-0.7Ta-1.2O alloy composition exhibiting multifunctional properties [41]. Besides the above-mentioned properties, the increased biocompatibility in terms of corrosion resistance [42] and cellular response [42,43] recommend this alloy as an ideal candidate for biomedical applications, particularly, bone regeneration. Multicomponent Ti-GUM metal used in this study contains high Nb alloying element that acts as optimum β stabilizers and can form Nb_2_O_5_, which proved to be beneficial for wear resistance because of the good lubricating property. Tantalum (Ta) is also suitable for β stabilization, contributing to the decrease of the modulus of elasticity and to the increase of the mechanical resistance, but it is substantially more expensive than Nb. Although, zirconium (Zr) is not a β-stabilizer, the addition of small quantities to Ti–Nb alloys to obtain ternary Ti–Nb–Zr, induces the behavior of Zr as a β-stabilizer and suppresses the formation of the ω martensitic precipitate [18].

Like other classes of Ti alloys, the functional properties of GUM metals are structure-sensitive. Nanostructured Ti alloys with a grain size below 1000 nm may improve the materials’ mechanical behavior and the biological response when compared to their coarse-grained material counterparts [40]. An effective manner to fabricate bulk nanostructured metallic materials is severe plastic deformation (SPD) [40,44]. In the last years, numerous investigations have been performed to develop and explore various methods for the SPD processing of the materials [45,46,47]. Among these methods, the equal-channel angular extrusion (ECAE) [40,48] is the most studied and largely used technique to date. Another frequently used technique that has been proved to be promising for ultrafine structure formation in the metallic alloys is high pressure torsion (HPT) [40,49]. During HPT technique, the deformation occurs under the action of hydrostatic pressure and shear strain. Out of several HPT methods, high speed high pressure torsion (HSHPT) is a recently developed SPD technique that has been successful in obtaining the bulk ultrafine-grained (UFG) alloys [50,51,52]. The grain size refinement mechanism during HSHPT is determined by extremely high shear strains within the workpieces [52]. This technique is one of the most effective for achieving grain refinement by combining high hydrostatic compressive stresses with torsion induced through high rotation speed of the superior punch. The imposition of high hydrostatic pressure during the process drives to a high amount of plastic strain. On the other hand, the high torsion revolution of 1795 rpm (as against 1 rpm specific to HPT), led to heating by friction of the workpiece inside the machine [51]. Thereby, the accomplishment of the HSHPT technology requires a short period of time, just a few seconds. Another advantage is that this method can be used to make larger sized components when compared with the classical HPT.

The aim of the present study is to examine the effects of HSHPT severe plastic deformation in conjunction with CR deformation on the evolution of the Ti-31.5Nb-3.1Zr-3.1Ta-0.9Fe-0.16O alloy microstructure and to assess the preosteoblast response to this newly developed GUM alloy. The final purpose is to establish the suitability of the HSHPT+CR-processed alloy for compression staples with the multidirectional grip, able to ensure a better fixation of bone fragments, especially in the case of multiple fractures.

## 2. Materials and Methods

### 2.1. Alloy Synthesis

Ingots of the GUM metal with new chemical composition Ti-31.5Nb-3.1Zr-3.1Ta-0.9Fe-0.16O (% mass) were produced by cold crucible induction in levitation melting, using a FIVE CELES MP25 furnace (Lautenbach, France). The synthesis was conducted under argon atmosphere using commercially high-purity raw metals: (i) titanium grade 3, with 0.30% Fe; 0.05% N_2_; 0.25% O; max. 0.013% H; 0.10% C; balance Ti—from ZIROM, Giurgiu, Romania; (ii) niobium (Nb) 99.81% having 0.005% Fe; 0.005% Si; 0.010% Mo; 0.010% W; 0.002% Ti; 0.002% Cr; 0.1% Ta; 0.005% Ni; 0.02% O; 0.02% C; 0.0015% H; 0.015% N—from Treibacher Industrie AG, Althofen, Austria; (iii) tantalum (Ta) with 99.95% Ta, having 0.03% Nb; 0.0022% C; 0.0093% O; 0.001% Co; 0.20% Si; 0.001% Mo; 0.0047% other—from Alfa Aesar (Thermo Fisher, Kandel, Germany); (iv) zirconium (Zr) 99.6% having 0.01% Fe; 0.035% Si; 0.03% Mo; 0.05% W; 0.01% Ti; 0.02% Ni; 0.02% O; 0.01% C; 0.0015% H; 0.01% N; 0.2% Nb; balance Zr—from ZIROM, Giurgiu, Romania and (v) iron (Fe), having 0.015% C; 0.01% Si; 0.02% Mn; 0.02% S; 0.01% P; 0.015% O; balance Fe—from Metarex, Bucharest, Romania. The oxygen (O) necessary to obtain the alloy is contained in the other elements of its composition (especially in Ti). Due to the significant differences in the melting point temperature of component elements (Ti: 1660 °C; Nb: 2468 °C; Zr: 1855 °C; Ta: 2996 °C; Fe: 1538 °C) and in density (Ti: 4.51 g/cm^3^; Nb: 8.58 g/cm^3^; Zr: 6.51 g/cm^3^; Ta: 16.68 g/cm^3^; Fe: 7.87 g/cm^3^) the melting may hinder. To ensure a high degree of compositional homogeneity the ingots were melted twice. Afterwards, the ingots were mechanically machined and cut in disk-shaped pieces having the diameter of 18.5 mm and the height of 7–8 mm.

### 2.2. HSHPT- and CR-Processing of the GUM Alloy

The disks cut from the GUM metal ingots were subjected to the HSHPT technology with an applied pressure p of about 1 GPa and different logarithmic deformation degree between 0.68 and 2.02. For HSHPT to which the torsion is induced through high rotation speed of the superior punch (975 rpm) it is suitable to use the true logarithmic degree strain (ε) determined as follows [53]:

(1) $$\varepsilon \;=\;\ln\frac{h_{0}}{h}$$

where *h*_0_ is the initial height of the sample and h depicts the final thickness of the sample.

HSHPT technology is designed to produce fine and ultrafine structures starting from as-cast alloys or hard to deform alloys. Thin 3D shells with ultrafine structure exhibiting diameters up to 45 mm or thin disks can be obtained by this method. In the HSHPT process a high pressure of 1–2 GPa is applied on the lower anvil while the upper anvil is driven with a rotation speed, which varies between 10^2^ and 2 × 10^3^ rpm. A large slippage between anvils and samples takes place determining a great amount of heat generated by intense friction. The hot SPD occurs very fast, taking only 10–20 s. The thin samples expand on a large cold area of the anvils and the temperature decreases rapidly by thermal conduction. The recrystallization is limited so that at the room temperature the samples show an ultrafine-grained structure [54].

As-processed HSHPT specimens incorporating 0.68 degrees of deformation were cold rolled using a homemade quattro rolling mill driven by a 5 kW electric motor. The rotational speed of the working cylinders was varied between 0 and 30 rpm using a power thyristor converter. The diameter of the working cylinders was 36 mm, which allowed one to obtain semi-finished products with a small thickness and a good surface quality. The rolling force was between 10 and 15 kN. The rolling was accomplished at a speed of 3 m/min, without the application of lubricants, the degree of deformation at one pass being approximately 10%, and the cumulative degree of deformation of 45%–72%. The strips processed by cold rolling were 1.5–0.8 mm in thickness. The logarithmic strain degree of samples obtained after HSHPT technology in conjunction with cold-rolling (CR) deformation was from 1.46 to 2.09.

The manufacturing process of the compression staples with multidirectional grip can be approached in two ways. The first one that can be adopted refers to the processing of thin hollow semi-finished products directly through HSHPT, followed by cutting the staples and their functionalization. In the second path, HSHPT-processed flat disks are cold rolled, cut, and functionalized. Therefore, the effects of severe plastic deformation by HSHPT and combining HSHPT with CR on the alloy structure and in vitro biocompatibility were monitored.

### 2.3. Alloy Samples’ Characterization

In order to investigate the microstructures of the developed GUM alloy in the as-cast state (GUM_as-cast), or after processing by HSHPT (GUM_HSHPT) and HSHPT plus CR (GUM_HSHPT+CR), the microscopy analysis was carried out using an “OLYMPUS BX51” (Tokyo, Japan) optical microscope with a video camera operated with the QCapture software package (QuickPHOTO MICRO 2.3, Prague, Czech Republic). A “Zeiss” (ZEISS EVO MA15, Munich, Germany) scanning electron microscope equipped with an analyzer for energy-dispersive X-ray spectroscopy was used to enable a better scrutiny of the dendritic microstructure. Prior to the microstructure analysis, the samples were successively subjected to mechanical polishing with different SiC abrasive papers up to grade 2000 and then finally polished with colloidal silica suspension (OP-S, Struers). The microstructure was disclosed by using a solution composed of 5% HF, 5% HNO_3_ and 90% H_2_O. The X-ray diffraction (XRD) analysis was carried out with a DRON 3 (Saint Petersburg, Russia) diffractometer, operated at 30 kV and 20 mA, at room temperature.

### 2.4. In Vitro Biocompatibility Assessment

#### 2.4.1. Cell Culture Model

The cell culture model used for in vitro biocompatibility studies was represented by murine preosteoblasts (MC3T3-E1 subclone 4, ATCC^®^, CRL-2593^TM^, Manassas, VA, USA). These cells were plated on the surface of the assayed metallic disks at an initial density of 8 × 10^3^ cells·cm^−2^ except for cell differentiation studies when 4 × 10^4^ cells·cm^−2^ were seeded. Afterwards, the cell-populated specimens were incubated in standard and osteogenic culture conditions, respectively, as reported in a previous recent paper [55]. The medium was exchanged every two days and all experiments have been performed in triplicate. Previously to cell plating, the disks were sonicated twice in 70% ethanol for 30 min to remove any particles resulting from the manufacturing process. Then, they were successively subjected to washing with sterile-filtered Milli-Q water, sterilization under ultraviolet light in a sterile tissue culture hood for 1 h on each side, and conditioning in culture medium.

#### 2.4.2. Microscopic Evaluation of MC3T3-E1 Cell Adhesion and Morphology

The adhesion and morphological characteristics of MC3T3-E1 cells grown in contact with each sample were investigated in fluorescence microscopy by vinculin and actin labeling, after 2 h and 24 h of culture, as previously described [56]. Briefly, the cells fixed with a cold solution of 4% paraformaldehyde in phosphate-buffered saline (PBS) and permeabilized with a solution containing 2% bovine serum albumin (BSA) and 0.1% Triton X-100 in PBS were sequentially incubated with mouse monoclonal anti-vinculin IgG1 (Santa Cruz Biotechnology, Dallas, TX, USA) and goat anti-mouse IgG antibody conjugated with Alexa Fluor 546 (Invitrogen, Eugene, OR, USA). Then, the actin cytoskeleton and the nuclei were successively labeled by treatment with Alexa Fluor 488 phalloidin (Invitrogen, Eugene, OR, USA) and 4′,6-diamidino-2-phenylindole (DAPI, Sigma-Aldrich Co., Steinheim, Germany), respectively. After washing with PBS, the labeled specimens were visualized under an inverted microscope (Olympus IX71, Olympus, Tokyo, Japan) and the fluorescent images were taken by using the Cell F image acquiring system (Version 5.0, Olympus Soft Imaging Solutions, Münster, Germany). The number of focal adhesions per cell were evaluated by means of the Image J software, as presented in the same paper [55]. A freehand selection tool was used to analyze the representative pictures of vinculin fluorescent staining following their conversion to grayscale images and the background subtraction. After the threshold adjustment the focal adhesions were counted using the analyze particles function.

#### 2.4.3. Assay of Cell Viability and Proliferation

The capacity of the cells to survive onto the samples was determined by preosteoblast fluorescence staining with the LIVE/DEAD Cell Viability/Cytotoxicity Assay Kit (Molecular Probes, Eugene, OR, USA), as we reported in a previous paper [56]. In brief, at 1, 3 and 5 days after cell seeding, the preosteoblasts in contact with the surface of the metallic disks were incubated in calcein-AM (2 mM) and ethidium homodimer-1 (4 mM) solution and after washing with PBS they were investigated with an inverted microscope Olympus IX71 to discriminate between the viable cells (green fluorescence) and dead cells (red fluorescence). At the same time points, the proliferation potential of the MC3T3-E1 cells was quantified by their capacity to reduce WST-8 (2-(2-methoxy-4-nitrophenyl)-3-(4-nitrophenyl)-5-(2,4-disulfophenyl)-2H-tetrazolium, monosodium salt) compound to an orange soluble formazan that was measured at 450 nm [55] by using an automatic multi-well microplate reader (FlexStation 3 Multi-Mode Microplate Reader, Molecular Devices, San Jose, CA, USA).

#### 2.4.4. Evaluation of the MC3T3-E1 Preosteoblast Differentiation

Osteogenic differentiation of the MC3T3-E1 cells was evaluated by quantifying alkaline phosphatase (ALP) activity and collagen synthesis under osteogenic culture conditions (supplementation of standard culture medium with 50 μg/mL ascorbic acid (Sigma-Aldrich Co., St. Louis, MO, USA), 5 mM β-glycerophosphate (Sigma-Aldrich Co., St. Louis, MO, USA) and 10^−8^ M dexamethasone (Sigma-Aldrich Co., St. Louis, MO, USA)). After 7 and 14 days of culture, the ALP enzymatic activity was measured in cell lysates by using the Alkaline Phosphatase Activity Colorimetric Assay Kit (BioVision, Milpitas, CA, USA), as we presented in a previous study [57]. In summary, after incubation of the cell lysates with p-nitrophenyl phosphate (pNPP) solution at an alkaline pH, the OD values of the reaction product p-nitrophenol (pNP) were measured at 405 nm using a multiwell microplate reader (FlexStation 3 Multi-Mode Microplate Reader, Molecular Devices, USA). In parallel the protein concentrations were determined by means of the Bradford reaction and ALP activity was normalized to 1 µg of protein.

Collagen synthesis and deposition on the disks’ surfaces were quantified after 2 weeks of incubation in the osteogenic culture conditions by staining with Sirius Red, as previously described [43]. Briefly, the cell-populated specimens were fixed with 10% paraformaldehyde and then successively subjected to three washes with deionized water, incubation in Sirius Red Solution 0.1% (Bio-Optica, Milano, Italy), other washes and air drying. The OD of the colored solution resulted after solubilization in 0.2 M NaOH/methanol (1:1) was recorded at 540 nm.

#### 2.4.5. Statistical Analysis

Statistical analysis was conducted by means of the GraphPad Prism software (Version 3, GraphPad, San Diego, CA, USA) using a one-way ANOVA with Bonferroni’s multiple comparison tests. The obtained data are shown as mean ± SD (standard deviation). The *p* values below 0.05 were regarded as statistically significant.

## 3. Results and Discussion

### 3.1. Optical Microstructure of the GUM Alloy in As-Cast, HSHPT- and HSHPT+CR-Processed States

It is widely known that β Ti alloys are considered an attractive choice for orthopedic implants due to their excellent corrosion resistance, high biocompatibility, and low mutagenicity supposed to be safe for humans [1]. Among these GUM metals, called the second generation of Ti alloys, a new chemical composition was obtained, and a new deformation route was applied in the present study. The high refinement of the microstructure was induced in the first step by the HSHPT severe plastic deformation method. Further on, the HSHPT-processed disks were cold rolled so as to fabricate large workpieces from which to cut packages of orthopedic clamps

**Figure 1:**
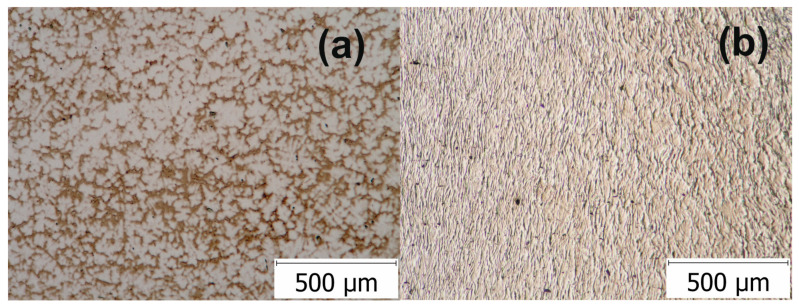
Optical microscopy images showing the microstructure of the Ti-Nb-Zr-Ta-Fe-O alloy in the: (**a**) as-cast and (**b**) high speed high pressure torsion (HSHPT) processed states.

Figure 1 presents morphological aspects of the alloy microstructure before and after the first step of plastic deformation. In the as-cast state, the Ti-31.50Nb-3.10Zr-3.10Ta-0.90Fe-0.16O (% mass) GUM alloy shows an inhomogeneous β solid solution with dendritic grains, as shown in Figure 1a. The optical microscopy image of the investigated GUM metal after the HSHPT process exhibiting a 0.68 logarithmic degree of deformation shows a high grain refinement and spatial redistribution of the β phase (Figure 1b). As observed, the HSHPT processed material revealed an almost homogeneous microstructure as compared to the initially dendritic microstructure. The microstructural refinement of β phase highly deformed was obvious. Additionally, one may observe a homogeneous distribution of grain sizes.

**Figure 2:**
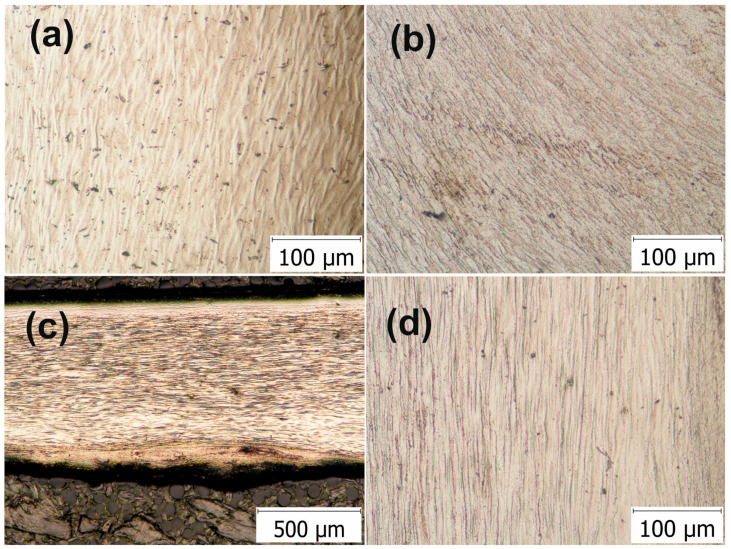
Optical microscopy images showing the microstructure of the Ti-Nb-Zr-Ta-Fe-O alloy subjected to HSHPT with: 0.68 (**a**), 1.3 (**b**) and 2.02 (**c**,**d**).

The HSHPT processed specimens with heavy deformation demonstrated significant refinement (Figure 2). The microstructural observations regarding the aspects of the microstructure and morphology of the HSHPT processed alloy predominantly show the presence of the highly deformed and textured β phase. By comparing the changes in the microstructure as the degree of deformation increased, one might observe that the microstructure densified. Additionally, the texture supported changes. At a low degree of deformation, a rather equiaxed winding relief was present (Figure 2a). In the peripheral area of the disk subjected to ε = 1.3, a curved and narrower flow line stands out (Figure 2b). Above ε = 2, the GUM metal shows finer and elongated flow lines in the transversal direction (Figure 2c,d).

It is known that fatigue resistance varies with the alloy microstructure [40]. Formation of ultrafine-grained range in staples is of great interest because it can play an important contribution to the improvement of mechanical and functional properties [58,59]. Ultrafine-grained (UFG) materials consist of grains with a size in a submicron (<1 µm) or nanocrystalline (<100 nm) range [60]. Regardless of the grain boundaries in conventional polycrystalline materials, the UFG materials contain in their microstructure a very high density of grain boundaries. Such a density of grain boundaries with peculiar features exerts a notable influence on the properties and mechanical behavior of the bulk nanostructured alloys.

**Figure 3:**
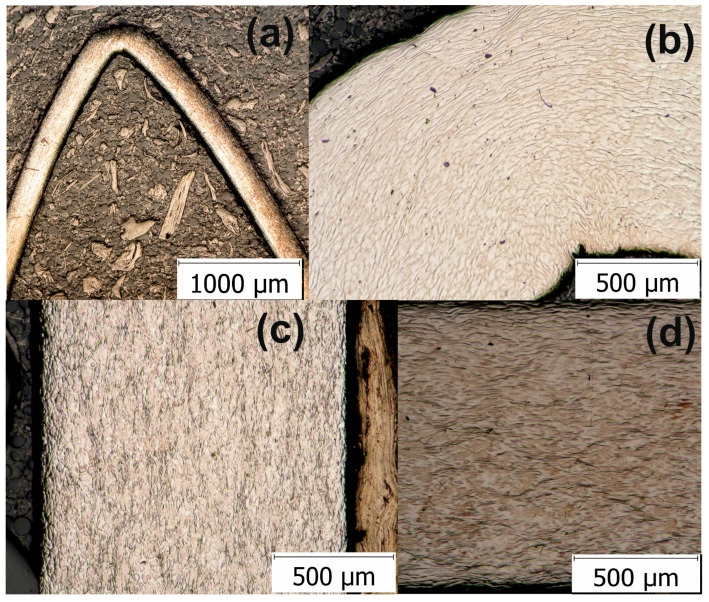
Optical microscopy image of the GUM metal: (**a**) orthopedic staple, (**b**) bending area of the staple, (**c**) HSHPT+CR processed clamp in the longitudinal rolling direction and (**d**) in the transverse rolling direction.

**Figure 4:**
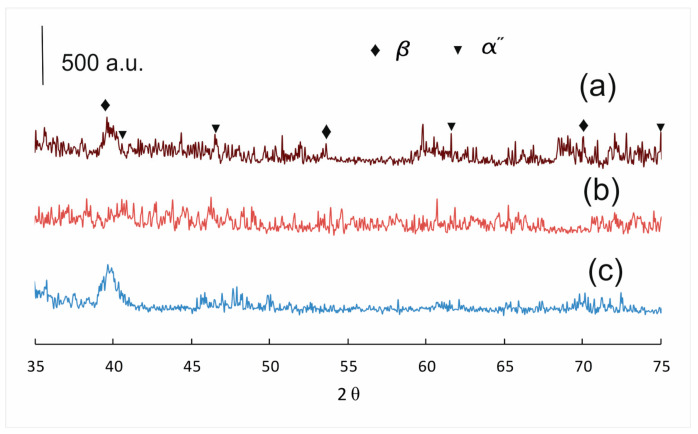
XRD patterns of the Ti-Nb-Zr-Ta-Fe-O alloy in: (**a**) as-cast, (**b**) HSHPT- and (**c**) HSHPT+CR- processed states.

HSHPT-processed specimens incorporating the 0.68 deformation degree were cold rolled using multi-step cross-rolling (the sample was turned 90° after each rolling pass). Cold rolling applied on severely plastic deformed disks provides additional large workpieces for designed new compression staples with the multidirectional grip. The final thickness of each sample was achieved in 23–27 passes. For each rolling pass the reduction of the sample thickness was 0.2 mm. Following HSHPT and cold rolling (CR), the samples were cut and banded for achieving orthopedic clamps. To gain a deeper understanding of the correlation between cold-rolling and bending to the clamps’ microstructure, optical micrographs were taken (Figure 3). The aspect of the clamp’s microstructure was fine and elongated (Figure 3a). In the area of curvature can be observed the compression of fibers inside and stretching outside (Figure 3b). Figure 3c,d shows the micrographs of the samples subjected to combined HSHPT and CR in 23 passes. The optical microscopy was performed on the rolling surface (RD) and cross-sectional plane (TD) of the CR processed strip. As it is expected, the morphologic aspects of the specimens processed by HSHPT plus CR are different from those of the HSHPT-processed specimens. The micrograph of RD plane shows equiaxed fine grains while the TD plane presents elongated and narrower grains. The whole volume of the specimen on the RD plan was filled with continuous recrystallized grains, as seen in Figure 3c [61]. In the CR condition, this alloy presents not only the β phase but also stress induced phase changes determined by the tensile deformation [62]. This trend is correlated with the XRD patterns recorded in the RD plane (Figure 4c).

### 3.2. SEM-EDX Analysis of the GUM Alloy in the As-Cast State

The results of the typical energy-dispersive X-ray spectroscopy (EDX) spot analyses performed on the cross-section of the initial as-cast state sample are shown in Figure S1. EDX point analysis was performed in two of the areas belonging to the dendritic microstructure. In the dark region (point 1) it can be observed that the atomic percentage of Ti, Nb and Ta occurs almost in the expected ratio, just slightly lower, as compared with the chemical composition of the alloy. However, the Zr content is concentrated in these areas. This indicates a significantly increased content, reaching values of up to 22% (as against the alloy content of 3.1%). The bright region (point 2) was depleted in Zr and the content in Ti and Ta was increased. In the bright area of the cross section (predominantly in the volume of the specimen), Ti, Ta and Fe are distributed rather uniformly throughout the volume of sample (Figure S2). The Zr and Nb elements’ distribution profile shows a negative correlation. The as-cast specimens exhibited a typical dendritic microstructure, which is respectively a chemically inhomogeneous solid solution. The development of non-equilibrium microstructures was due to the subcooling conditions. These specimens were directly HSHPT processed without the preliminary homogenization treatment. After HSHPT deformation, the resulting structure consists of an ultrafine and chemical homogeneous microstructure.

### 3.3. XRD Analysis of the GUM Alloy in As-Cast, HSHPT- and HSHPT+CR-Processed States

XRD study of the Ti-31.50Nb-3.10Zr-3.10Ta-0.90Fe-0.16O alloy in the as-cast state and after processing by HSHPT and HSHPT plus CR highlights mainly spectral lines for the β phase (Figure 4). In the severely plastic deformation state after applying a 2.02 degree of deformation by HSHPT, the peaks were broadening (FWHM parameter), suggesting a decrease in the grain-size phase. The XRD spectra disclose a fine grain size. Moreover, due to internal stress caused by concomitant compression and torsion, the peaks were slightly shifted to right position. After processing by HSHPT plus CR, the peaks’ widths decreased pointing to the formation of equiaxed grains, most probably by continuous recrystallization.

### 3.4. In Vitro Cell Responses

#### 3.4.1. Cell Adhesion and Morphology

**Figure 5:**
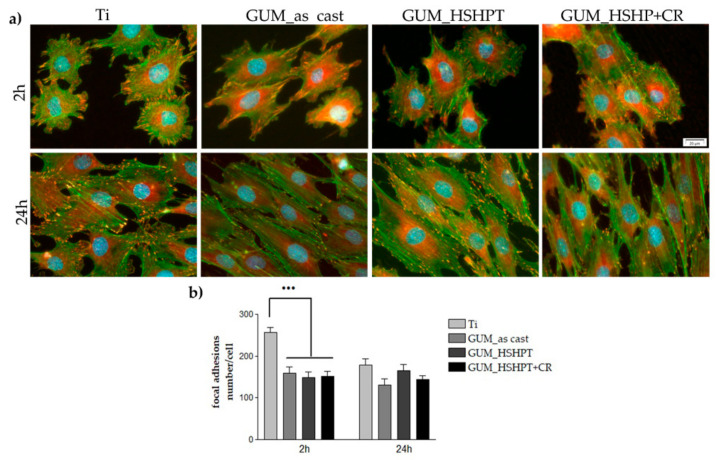
Fluorescent images of MC3T3-E1 preosteoblasts grown on tested substrates for 2 and 24 h, respectively. (**a**) Green fluorescence: actin cytoskeleton; red fluorescence: vinculin signals. Scale bar: 20 mm; (**b**) quantification of focal adhesions. Results are presented as means ± SD (*n* = 15 cells); ••• *p* ˂ 0.001 for GUM as-cast; GUM_HSHPT; GUM_HSHPT+CR vs. Ti at 2 h post seeding.

**Figure 6:**
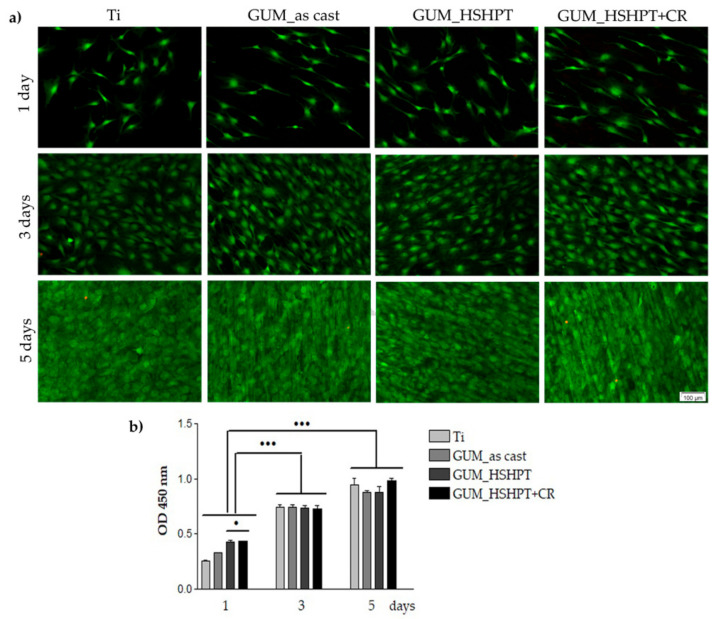
Viability and proliferation potential of MC3T3-E1 cells grown in contact with Ti, GUM in the as-cast state, GUM_HSHPT and GUM_HSHPT+CR surfaces for 1, 3 and 5 days. (**a**) Fluorescence microscopy images of the MC3T3-E1 cells stained with the LIVE/DEAD Cell Viability/Cytotoxicity Assay Kit (live cells: green fluorescence; dead cells: red fluorescence). Scale bar: 100 µm; (**b**) results of the CCK-8 assay showing the proliferation ability of the cells grown on the analyzed surfaces (*n* = 3, mean ± SD). • *p* ˂ 0.05 for GUM_HSHPT and GUM_HSHPT+CR substrates vs. Ti at 1 day post-seeding; ••• *p* ˂ 0.001 for Ti, GUM alloy in the as-cast state, GUM_HSHPT and GUM_HSHPT+CR samples after 3 and 5 days of culture vs. 1-day incubation period.

It is well known that the primary interaction of the cells with the materials’ surface will impact on their further destiny. Thus, in the present study, the cellular response to the newly developed GUM alloy was firstly investigated by the concomitant immunofluorescent labeling of vinculin and actin cytoskeleton. Consequently, cell attachment, spreading and morphology were investigated in view to demonstrate the biocompatibility of the GUM alloy substrate with modified structure as compared to the bare one and the commercially pure titanium (Ti, 99.6% pure, ADVENT) considered as reference biomaterial. Vinculin, a key protein present in the focal contacts [63] that is involved in the process of cell adhesion by direct binding of actin, stimulating its polymerization and the recruitment of the actin remodeling proteins [64], provides a valuable detection system for focal adhesion sites using a specific antibody. After 2 h of cell culture, MC3T3-E1 preosteoblasts were attached to all surfaces and exhibited a predominant cortical actin distribution and numerous microextensions, such as lamellipodia and filopodia. Additionally, well expressed vinculin signals have been detected at the cell periphery (Figure 5a) that suggest the development of focal contacts at the interface between plasma membrane and extracellular proteins adsorbed to the materials’ surfaces. Quantification of focal contacts at this time point revealed the presence of a significantly higher (*p* < 0.001) number of vinculin positive signals on the Ti surface as compared to the bare and modified GUM alloy substrates (Figure 5b). Considering that the GUM alloys display a lower Young’ modulus than Ti, the results presented here demonstrate the link between the formation of focal contacts and changes in substrate stiffness [65]. This finding is consistent with the data presented by Zhang et al. [66] revealing that the expression levels of vinculin diminished as the substrates became softer. At 24 h post-seeding, fluorescence images presented in Figure 6a displayed the specific aspects of osteoblastic cells with well-organized actin stress fibers oriented parallel to one another and along the cell body, and mature vinculin signals at their termini. Additionally, no significant differences (Figure 5b) with respect to the formation of focal contacts could be observed between analyzed samples at this time point. Our findings are in trend with the results reported by Gordin et al. [43] showing no significant differences concerning the cell spreading, the formation of focal contacts and cytoskeleton organization between the Ti–23Nb–0.7Ta–2Zr–1.2O GUM alloy and Ti control sample over the culture period.

#### 3.4.2. Preosteoblast Viability and Proliferation Status

The capability of the developed GUM alloy specimens to sustain the survival and proliferation of MC3T3-E1 cells seeded on their surfaces was investigated through labeling of living and dead cells by means of the LIVE/DEAD Viability/Cytotoxicity kit and through quantifying the WST-8 reduction by mitochondrial dehydrogenases from metabolically active viable cells to a water-soluble formazan. Figure 6a shows fluorescent images taken from cell populated surfaces after performing the LIVE/DEAD assay. At 1-day post-seeding, all preosteoblasts grown in contact with the tested alloy surfaces exhibited green fluorescence, which is a characteristic of the viable cells. By prolonging the cell incubation period up to 5 days, a progressive increase in cell number is visible and no red dead cells could be observed except for a few cells that appeared after 5 days of culture due to the occurrence of contact inhibition at very high cell density. These discoveries suggest the absence of any considerable cytotoxicity against the MC3T3-E1 cells. Further on, the proliferation of the preosteoblasts in contact with the developed materials was examined by performing the CCK-8 assay. As presented in Figure 6b, the metabolic activity (denoted by OD values) of the MC3T3-E1 preosteoblasts on the tested surfaces increased gradually from 1 day to 5 days of culture (*p* < 0.001 at 3 and 5 days post-seeding vs. 1-day incubation period). Moreover, no significant differences in the cell proliferation status were noticed among the tested samples with the exception of the 24 h-time point when a significant increase in the OD value (*p* < 0.05) was recorded for GUM_HSHPT and GUM_HSHPT+CR samples in comparison to the GUM alloy in the as-cast state and the control surface. Altogether, these data demonstrate that all investigated materials encourage cell proliferation without showing any deleterious effect revealing their enhanced biocompatibility. Altogether, these results are consistent with a prior study performed by Xue et al. on the new Ti-19Zr-10Nb-1Fe alloy exhibiting a low Young’s modulus (59 GPa), which demonstrated that MC3T3-E1 preosteoblasts possess almost similar capacity to adhere and proliferate on this superelastic alloy and NiTi alloys [67]. Likewise, our previous experiments showed that MC3T3-E1 cells exhibited the same ability to attach and grow on the novel superelastic beta-type Ti–23Nb–0.7Ta–2Zr–0.5N alloy and the reference material, i.e., Ti–6Al–4V [29]. Moreover, the bare and nitrided Ti-23Nb-0.7Ta-2Zr-0.5N alloy did not exert harmful effects on the cells, and sustained the adhesion and enhanced proliferation of MG63 osteoblasts as compared to the commercially pure Ti [32,68].

#### 3.4.3. The Differentiation of MC3T3-E1 Preosteoblasts

**Figure 7:**
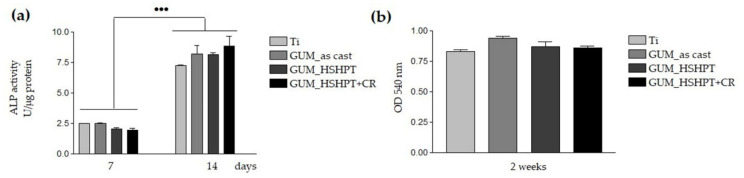
Differentiation of the MC3T3-E1 cells cultured in contact with the Ti and GUM alloy in the as-cast state and GUM_HSHPT and GUM_HSHPT+CR substrates, as assessed by: quantification of ALP activity at 7 and 14 days post-seeding (**a**) and determination of the collagen synthesis after 2 weeks of culture (**b**). Data analysis was based on mean ± SD (*n* = 3). ••• *p* ˂ 0.001 for ALP activity after 14 days of culture vs. ALP activity expressed at 7 days post-seeding.

The potential of the developed materials to promote the preosteoblast differentiation under osteogenic culture conditions was investigated by studying the expression of the osteoblast specific markers like ALP and collagen synthesis, as compared to the Ti reference biomaterial. ALP is an enzyme of glycoprotein nature that is prevalently expressed in many tissues, including the bone tissue, where it plays a crucial role in its mineralization [69]. It is found both bound to the surfaces of osteoblasts through a phosphoinositol linkage and free inside the mineralized bone matrix [70]. Mineralization of diverse types of tissue, such as bone, teeth and cartilage, occurs in multiple physicochemical and biochemical processes that stimulate the mineralization process by the deposition of hydroxyapatite-based microcrystals into collagen fibrils of the extracellular matrix [71]. The enzymatic activity of ALP, an early marker of osteogenic differentiation, was measured after 7 and 14 days of culture. The graph presented in the Figure 7a shows a highly significant increase of ALP activity (*p* < 0.001) for all studied samples after 14 days of culture as compared to the 7–day incubation period. This increase relieves the stimulating effect of all analyzed specimens on the MC3T3-E1 preosteoblast differentiation toward an osteogenic lineage. To note that for both experimental times no statistically significant differences between these samples were detected.

Further on, we investigated another osteogenic marker, i.e., collagen matrix synthesis by quantifying collagen deposition following staining with Sirius Red after 2 weeks of culture of the MC3T3-E1 preosteoblasts grown in contact with the analyzed surfaces (Figure 7b). Type I collagen represents the most ample protein inside the bone extracellular matrix (ECM) functioning as a scaffold for mineral depositions. Besides, this fibrillar molecule binds to and orients other matrix proteins involved in the mineralization process. It also binds to the integrin molecules crossing the plasma membrane and initiates the signaling pathway that activates Runx2 (Runt-related transcription factor 2) known to be responsible for osteoblast differentiation and expression of other proteins specific to bone ECM, like osteopontin, bone sialoprotein, osteocalcin, etc. [72,73,74]. The graph presented in the Figure 7b reveals that all tested metallic surfaces possessed the same potentials to support the preosteoblast differentiation.

## 4. Conclusions

In the present work, in vitro biological performance of the newly synthesized Ti-31.5Nb-3.1Zr-3.1Ta-0.9Fe-0.16O GUM alloy that was subjected to the severe plastic deformation process via HSHPT followed by CR (GUM_HSHPT+CR) was comparatively investigated with the GUM alloy in the as-cast state and GUM_HSHPT substrate. The results were also related to the reference biomaterial, namely commercially pure titanium. Fabrication by HSHPT and CR processes of the developed GUM alloy demonstrated the capability of decreasing the grain size. The structural investigations showed that by increasing the deformation, a high density of grain boundaries is accumulated, leading gradually to the fine grain size. Cell culture experiments conducted with the MC3T3-E1 cell line proved that all studied surfaces favor the cell adhesion, survival and proliferation and osteogenic differentiation without showing any deleterious effect. Therefore, the severe plastic deformation by HSHPT technology endows the new GUM alloy with structural features and increased biocompatibility required for developing promising bone compression staples.

## Supplementary Materials

The following are available online at https://www.mdpi.com/1996-1944/13/21/4853/s1, Figure S1: SEM-EDX point analysis of the as-cast sample: point 1-dark area and point 2-bright area of dendritic structure; Figure S2: Line scan by EDX across the full thickness of the as-cast sample highlighting the variation of its chemical composition.
