# Supplementary material for: The Influence of Severe Plastic Deformation on Microstructure and In Vitro Biocompatibility of the New Ti-Nb-Zr-Ta-Fe-O Alloy Composition
Source: Materials (Basel). 2020 Oct 29;13(21):4853. doi: 10.3390/ma13214853 (PMC7663053; doi:10.3390/ma13214853)
Supplement: Supplementary file 1 [file materials-13-04853-s001.pdf]

materials

The Influence of Severe Plastic Deformation on Microstructure and in Vitro Biocompatibility of the New Ti-Nb-Zr-Ta-Fe-O Alloy Composition

Carmela Gurau [1], Gheorghe Gurau [1], Valentina Mitran [2], Alexandru Dan[3] and Anisoara Cimpean [2,*]

[1] Faculty of Engineering, "Dunărea de Jos" University of Galati, Domnească Street 47, 800008 Galati, Romania; carmela.gurau@ugal.ro (C.G.); gheorghe.gurau@ugal.ro (G.G.)
[2] Department of Biochemistry and Molecular Biology, University of Bucharest, 91-95 Splaiul Independentei, 050095 Bucharest, Romania; valentinamitran@yahoo.com
[3] R&D Consultanta si Servicii, 45 Maria Ghiculeasa, 020943 Bucharest, Romania; alexandru_dan_ro@yahoo.com
* Correspondence: anisoara.cimpean@bio.unibuc.ro; Tel.: +40-21-318-1575 (ext. 106)

Supplementary Materials

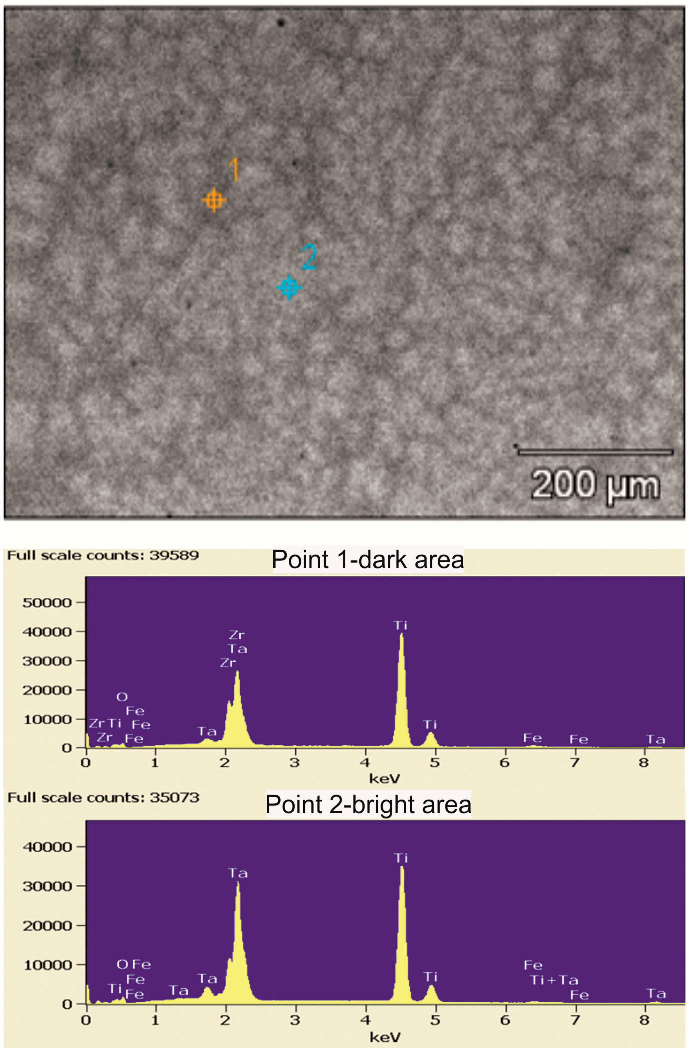

Figure S1. SEM-EDX point analysis of the as-cast sample: point 1-dark area and point 2-bright area of dendritic structure.

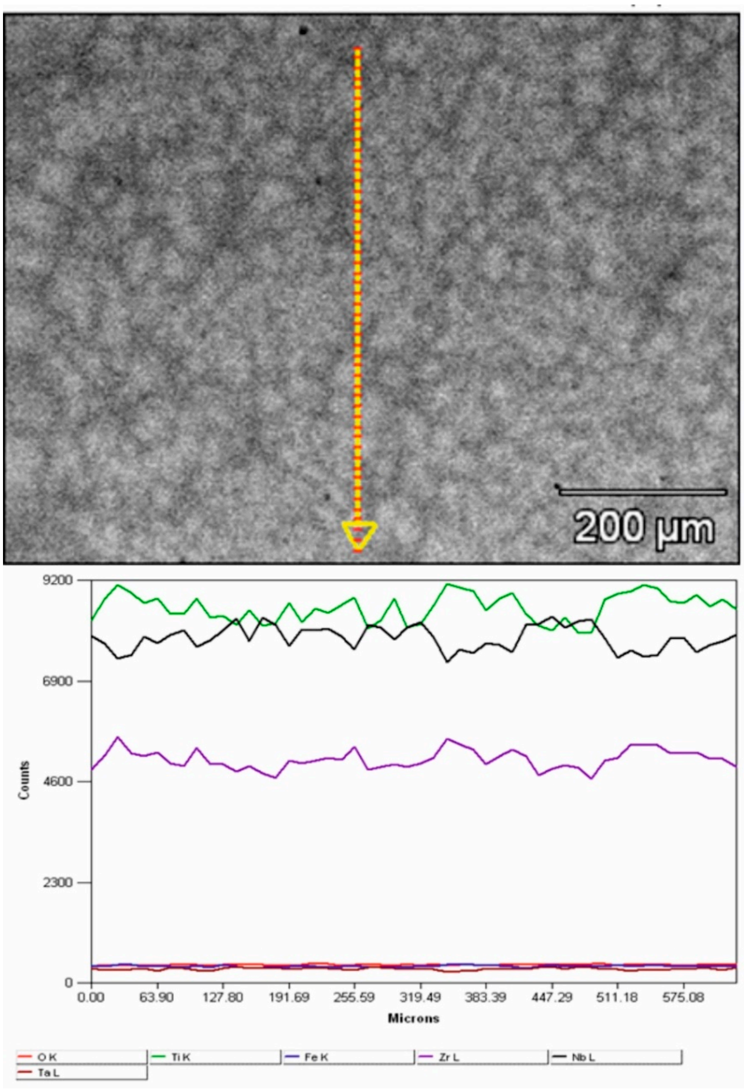

Figure S2. Line scan by EDX across the full thickness of the as-cast sample highlighting the variation of its chemical composition.
